# Chronic Nasal Catarrh

**Published:** 1885-02

**Authors:** 


### Chronic Nasal Catarrh.

The diagnosis and treatment of chronic nasal catarrh, embracing three clinical lectures, delivered at the College of Physicians and Surgeons, New York, by Geo. M. Lefferts, A.M., M.D. This is a valuable little *brochure* upon this subject.

Nasal catarrh is so intimately connected with the oral cavity and exercises so much influence upon its secretions and conditions that it should be studied and thoroughly understood by the dentist. He should be able, not only to recognize its presence, but to comprehend its different pathological phases, but the best methods of treatment.

The little work here referred to gives a large amount of information on these subjects, and well condensed, too. It may be obtained of Lambert & Co., St. Louis, Mo.

